# Enhanced rich club connectivity in mild or moderate depression after nonpharmacological treatment: A preliminary study

**DOI:** 10.1002/brb3.3198

**Published:** 2023-09-07

**Authors:** Zhiliang Long, Danni Chen, Xu Lei

**Affiliations:** ^1^ Sleep and NeuroImaging Center Faculty of Psychology Southwest University Chongqing P. R. China; ^2^ Key Laboratory of Cognition and Personality (Southwest University), Ministry of Education Chongqing P. R. China

**Keywords:** depression, functional MRI, nonpharmacological treatment, rich club

## Abstract

**Introduction:**

It has been suggested that the rich club organization in major depressive disorder (MDD) was altered. However, it remained unclear whether the rich club organization could be served as a biomarker that predicted the improvement of clinical symptoms in MDD.

**Methods:**

The current study included 29 mild or moderate patients with MDD, who were grouped into a treatment group (receiving cognitive behavioral therapy or real‐time fMRI feedback treatment) and a no‐treatment group. Resting‐state MRI scans were obtained for all participants. Graph theory was employed to investigate the treatment‐related changes in network properties and rich club organization.

**Results:**

We found that patients in the treatment group had decreased depressive symptom scores and enhanced rich club connectivity following the nonpharmacological treatment. Moreover, the changes in rich club connectivity were significantly correlated with the changes in depressive symptom scores. In addition, the nonpharmacological treatment on patients with MDD increased functional connectivity mainly among the salience network, default mode network, frontoparietal network, and subcortical network. Patients in the no‐treatment group did not show significant changes in depressive symptom scores and rich club organization.

**Conclusions:**

Those results suggested that the remission of depressive symptoms after nonpharmacological treatment in MDD patients was associated with the increased efficiency of global information processing.

## Graphical abstract

1. The depressive symptoms were improved after nonpharmacological treatment. 2. Nonpharmacological treatment increased brain network functional connectivity. 3. The treatment response was related with enhanced rich club organization.

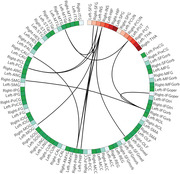

## 1 INTRODUCTION

The human brain is a complex functional system of interconnected regions which is called the human connectome (Sporns et al., 2005). Once the functional connections of the system are damaged or abnormal, information processing and integration will be impaired and eventually lead to brain dysfunction (van den Heuvel et al., 2013). Major depressive disorder (MDD), which is characterized by persistent depressed mood, anhedonia, difficulty in concentration, and a strong sense of meaninglessness (Belzung et al., 2015), has been hypothesized as a disorder of brain functional connectivity (FC). For example, neuroimaging studies report increased FC in the medial prefrontal cortex (Mulders et al., 2015), and posterior cingulate cortex (Dong et al., 2019) in patients with MDD. Furthermore, patients with MDD have brain dysfunction at the network level, mainly involving the default mode network (DMN), the central executive network, and the salience network (SN) (Meng et al., 2021; Mulders et al., 2015). Additionally, patients with MDD are involved in topological abnormalities of several brain regions (Yun & Kim, 2021) that have a central role in supporting integrated brain function.

Recently, it has been noticed that some brain regions have high degree, low clustering, short path length, and high centrality and participate in multiple brain region communities. These brain areas are named “brain hubs.” The hubs that are more closely connected than lower degree nodes are called “Rich Clubs” (van den Heuvel & Sporns, 2011). It plays an essential role in promoting global brain nerve signaling and regional interbrain communication and integration (van den Heuvel et al., 2012). Existing studies have confirmed that the rich club organization in depressed persons is damaged (Liu et al., 2021), which is considered a potential indicator to distinguish depressed patients from cognitively normal individuals.

At present, nonpharmacological treatment has been proven to be an effective method for the treatment of MDD, such as cognitive behavioral therapy (CBT) and neurofeedback training, but the brain mechanism underlying the treatment improvement remains unclear. Previous studies have linked improved outcomes to changes in specific FCs of brain networks. For example, CBT has been demonstrated to have antidepressant effects (Hollon et al., 2014) and normalize the FCs of the limbic system in depressed adolescents (Chattopadhyay et al., 2017). After CBT intervention, FCs of the amygdala and frontoparietal lobes in patients with MDD are significantly enhanced (Shou et al., 2017). Following neurofeedback therapy, functional hubs of the DMN, such as the medial prefrontal cortex and precuneus, are found to have increased FC with the amygdala (Young et al., 2018). Unfortunately, it is not clear whether the rich club organization is changed in MDD patients after nonpharmacological treatment.

Thus, the current study aims to explore whether the rich club organization is improved in patients with MDD after nonpharmacological treatment. Based on previous findings, we hypothesize that (1) nonpharmacological treatment enhances the rich club organization; (2) the improvement of rich club organization is correlated with the remission of clinical symptoms.

## 2 MATERIALS AND METHODS

### 2.1 Participants

The database used in the current study was obtained from the OpenNeuro dataset (https://openneuro.org/datasets/ds003007/versions/1.0.1) (Bezmaternykh et al., 2021). This study included 29 patients with mild or moderate depression (8 males, 21 females), who were scanned twice (pre‐ and post‐session) with 2–3 months intervals. These participants were classified into 2 groups: the treatment group including 14 patients who received a brief CBT course (8 participants, 3 males, and 5 females) or real‐time fMRI neurofeedback course (6 female participants), and the no‐treatment group including 15 patients (5 males, 10 females) who received no treatment. The exclusion criteria for all participants were (1) neurological or psychotic level mental disorders; (2) psychotropic medication or drugs; (3) contraindications to MRI; (4) bipolar, seasonal, or secondary to other disease; (5) intelligence quotient that was proven with Raven Progressive Matrices test less than 70. Several clinical scores, including Montgomery–Asberg Depression Rating Scale (MADRS), Beck Depression Inventory (BDI), Zung self‐rating depression scale (Zung‐SDS), Marlowe–Crowne Social Desirability Scale (MC‐SDS), Toronto Alexithymia Scale (TAS), and Experiences in Close Relationship Scale (ECR), were recorded both in pre‐ and post‐session. The detailed demographic information can be seen in Table 1.

**TABLE 1:** Demographic information of participants.

Variables	Treatment group (*n* = 14)		No‐treatment group (*n* = 15)	
Age (years, mean ± sd)	30.3 ± 8.68		35.2 ± 9.35	
Gender (male/female)	3/11		5/10	
IQ (mean ± sd)	104.7 + 12.7		100.7 + 15.2	
	Presession	Post‐session	Presession	Post‐session
MADRS (mean ± sd)	**28.7 ± 2.6**	**15.7 ± 5.4**	–	–
BDI (mean ± sd)	**25.9 ± 9.7**	**15.8 ± 10.5**	18.4 ± 11.2	13.9 ± 11.3
Zung‐SDS (mean ± sd)	51.5 ± 5.7	47.2 ± 7.9	44.6 ± 8.4	41.3 ± 8.2
MC‐SDS (mean ± sd)	10.6 ± 1.7	10.5 ± 2	12.1 ± 2.5	12.2 ± 2.4
TAS (mean ± sd)	69 ± 11.3	66.8 ± 16	69.2 ± 13.6	63.3 ± 12.1
ECR‐avoidance (mean ± sd)	47.7 ± 14.6	45 ± 13.7	53.4 ± 13.5	48 ± 22.5
ECR‐anxiety (mean ± sd)	60.2 ± 14.6	55.5 ± 16.3	**63.5 ± 22.4**	**48.9 ± 21.2**

*Note*: Scores with bold indicates significant (*p* < .05) difference between pre‐ and post‐session. Note that some demographic information was missed. Specifically, in treatment group, 9 participants had MADRS scores, 11 participants had BDI and ECR scores, 10 participants had Zung‐SDS and TAS score, 8 participants had MC‐SDS scores. In no‐treatment group, 14 participants had BDI, Zung‐SDS, MC‐SDS, and TAS score; 13 participants had ECR scores.

Abbreviations: BDI, Beck Depression Inventory; ECR‐anxiety, Experiences in Close Relationship Scale—anxiety; ECR‐avoidance, Experiences in Close Relationship Scale—avoidance; IQ, IQ scores on Raven's Progressive Matrices; MADRS, Montgomery–Asberg Depression Rating Scale; MC‐SDS, Marlowe–Crowne Social Desirability Scale; TAS, Toronto Alexithymia Scale; Zung‐SDS, Zung self‐rating depression scale.

All the participants signed the informed consent before the experiment. This study was in accordance with Helsinki Declaration and was approved by local ethic board of the Institute of Molecular Biology and Biophysics.

### 2.2 Image acquisition

Participants were scanned in the International Tomography Center, Novosibirsk, using a Philips 3T Ingenia scanner. The participants were asked to remain motionless, keep their eyes closed, and not think of anything. Functional images were obtained by using an echo‐planar imaging sequence with the following parameters: repetition time/echo time = 2500/35 ms, voxel size = 2 ×  2 ×  5 mm^3^, flip angle = 90°, 25 slices, and lasting 6 min scanning. The anatomical image was obtained by the T1w 3D turbo field echo method with repetition time of 75 ms, voxel size of 1 ×  1 ×  1 mm^3^, 181 slices, and axial scanning.

### 2.3 Functional imaging preprocessing

The preprocessing of resting‐state MRI was conducted by using the SPM12 software toolbox (https://www.fl.ion.ucl.ac.uk/spm/software/spm12/). The first five volumes were discarded due to the adaptation of participants to the scanning environment and the magnetization stabilization. The remaining volumes were then corrected for the time delay between slices and the motion movement. The movement along the *x*, *y*, or *z* direction or their rotation around each axis of all participants was less than 2 mm or 2°. The resulting images were normalized into MNI standard space by using a unified segmentation of anatomical images and then resampled into a 3 × 3 × 3 mm^3^ voxel size. Several covariates of no interest, including 24 motion parameters, mean white matter signals, and cerebrospinal fluid signals, were removed via a multiple regression model. Currently, there were still debates about global signal regression (Murphy & Fox, 2017). In the current study, we did not regress the global signal, as suggested by previous studies (Leaver et al., 2016; Zhang et al., 2018). The resulting images were then linearly detrended and filtered within the range of 0.01–0.08 Hz.

### 2.4 Functional connectivity network construction

The Anatomical Automatic Labeling (AAL) atlas (Tzourio‐Mazoyer et al., 2002) with 90 cortical and subcortical brain areas was used to construct the FC network. We excluded the cerebellum from the AAL atlas, because previous studies had demonstrated that rich club regions mainly located in cortical areas (van den Heuvel & Sporns, 2011; van den Heuvel et al., 2012). The subnetwork affiliation of each AAL region was determined by using a previous report (Table S1) (Long et al., 2019). The mean time course was computed across voxels within each region of interest (ROI) of the AAL atlas. The Pearson correlation analysis was employed to calculate the correlation coefficient between the time courses of each pair of ROI, resulting in 90 ×  90 FC matrices. A family‐wise error correction method (*p* < .05) was applied in these FC matrices to exclude weak and spurious correlations. Specifically, there remains little consensus on handling or interpreting negative correlation (Murphy & Fox, 2017), so the negative correlation value was excluded in the current study (Lin et al., 2019).

### 2.5 Overall connectome organization

Several global and local graph metrics were computed for the FC network of each participant. Global metrics included (1) overall connectivity strength, calculated as the sum of all connections in the brain network; (2) global efficiency, calculated as the average inverse shortest path length, reflecting the overall capacity for parallel information transfer and integrated processing; (3) small‐wordness, computed as the normalized clustering coefficient divided by the normalized shortest path length, reflecting the optimal balance between local specialization and global integration. In addition to global metrics, node‐specific graph metrics were also computed, including (1) nodal strength, calculated as the number of connections of a node; (2) nodal efficiency, calculated as averaged inverse shortest path length of a node with all other left nodes; (3) nodal betweenness, calculated as the number of shortest path length passing through a node. The network metrics were computed using the GRETNA toolbox (https://www.nitrc.org/projects/gretna/).

### 2.6 Rich club organization

Rich club organization was described in detail in previous studies (Collin et al., 2014; Harriger et al., 2012; van den Heuvel & Sporns, 2011; van den Heuvel et al., 2012). In brief, the rich club organization of a network is a set of high‐degree (rich) nodes of the network that are tightly interconnected, forming a densely connected core of nodes.

**FIGURE 1:**
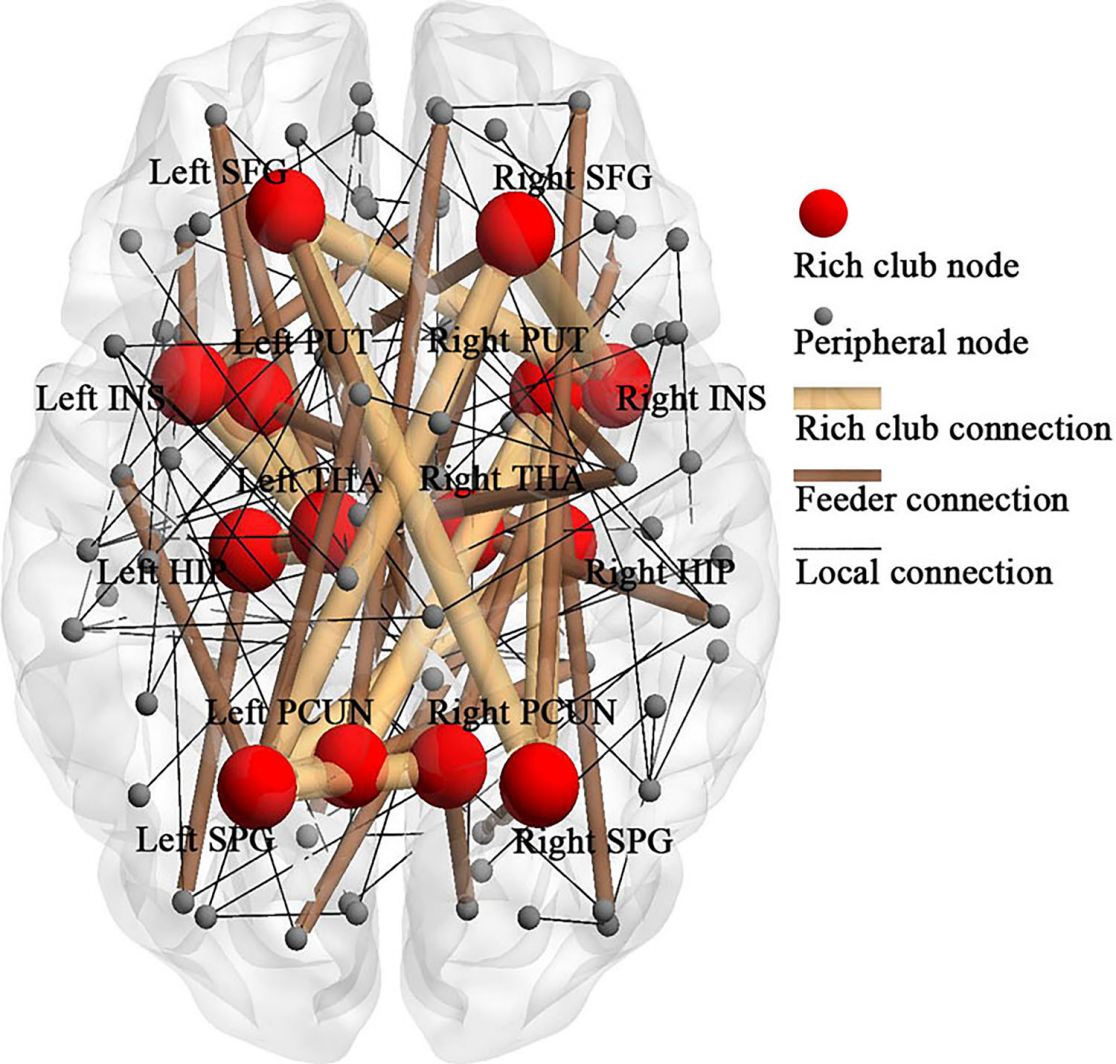
Schematic representation of rich club node, nonrich club peripheral node, rich club connection, feeder connection, and local connection. Fourteen nodes were identified as rich club nodes. HIP, hippocampus; INS, insula; PCUN, precuneus; PUT, putamen; SFG, superior frontal gyrus; SPG, superior parietal gyrus; THA, thalamus.

Considering that the current study did not include healthy participants, and the hub regions had been demonstrated to be altered in depression, thus the rich club regions were selected based on prior reports, as suggested by a previous study (Collin et al., 2014). In the current study, the rich club regions were defined as the following 14 brain areas: bilateral superior frontal gyrus, bilateral putamen, bilateral insula, bilateral thalamus, bilateral hippocampus, bilateral precuneus, and bilateral superior parietal gyrus (Figure 1). These rich club regions had well been validated by previous studies in human and nonhuman subjects (Collin, Sporns et al., 2014; Harriger et al., 2012; van den Heuvel & Sporns, 2011; van den Heuvel et al., 2012), which played crucial roles in pathophysiology of depression and were involved in nonpharmacological treatment improvement in this disease (Chattopadhyay et al., 2017; Liu et al., 2021; Meng et al., 2021). Following the definition, the nodes of network were classified into rich club nodes and peripheral (i.e., nonrich club) nodes.

Classification of rich club nodes allowed for the classification of the edges of network into (1) rich club connections, defined as connections between rich club nodes; (2) feeder connections, being the connections between rich club nodes and peripheral nodes; (3) local connections, which are the connections between peripheral nodes (Figure 1). For each participant, rich club connectivity, feeder connectivity, and local connectivity were computed as the sum of connectivity strength of rich club connections, feeder connections, and local connections, respectively.

Nonparametric Spearman's correlation analysis was employed to determine the linear relationship between rich club connectivity changes (post‐session minus presession) and changes of symptom score (post‐session minus presession), including MADRS, BDI, Zung‐SDS, and MC‐SDS. The statistical level of *p* < .05 was considered significant.

### 2.7 Statistical analysis

The differences of MADRS, BDI, Zung‐SDS, MC‐SDS, TAS, ECR‐avoidance, and ECR‐anxiety between presession and post‐session in the treatment and the no‐treatment groups were tested by using nonparametric two‐tailed Wilcoxon signed rank test (Table 1).

Two‐tailed Wilcoxon signed rank test was also employed to test the difference between pre‐ and post‐session in the treatment and the no‐treatment groups in the overall connectivity strength, global efficiency, small‐wordness, nodal graph metrics, rich club connectivity, feeder connectivity, and local connectivity. For the global metrics and rich club organization, the statistical level of *p* < .05 was considered significant. For the nodal metrics, a false discovery rate method of *q* < .05 was used to correct the multiple comparisons across nodes.

### 2.8 Network‐based statistics

Network‐based statistics (NBS) was used to identify which connected subnetworks were altered by the nonpharmacological treatment or natural disease progression in patients with depression. The NBS was implemented in the treatment and the no‐treatment groups separately. The procedure was as follows: Paired *t*‐test was used to test the difference of each connection between pre‐ and post‐session, resulting in *t* value of each connection. A primary threshold (here, *t* = 3) was applied to each connection, to define a set of suprathreshold connections. Any connected components or subnetworks within the set of suprathreshold connections were computed. The size of the components (number of edges) was then obtained. The null distribution of the component size was empirically obtained using a nonparametric permutation approach (5000 permutations) to estimate the significance of each connected component. A *p*‐value was assigned to each connected component by computing the proportion of component size exceeding the null distribution values. The subnetworks with *p*‐value less than .05 were considered significant.

## 3 RESULTS

### 3.1 Demographic difference

In the treatment group, there was a significant (*p* < .05) decrease in MADRS score, and BDI score after nonpharmacological treatment. The Zung‐SDS score, MC‐SDS score, TAS score, ECR‐avoidance score, and ECR‐anxiety score showed no difference (*p* > .05) between pre‐ and post‐session (Table 1). In the no‐treatment group, there was no difference in BDI score, Zung‐SDS score, MC‐SDS score, TAS score, or ECR‐avoidance score after natural disease progression, except for the ECR‐anxiety score which showed a significant decrease in post‐session (Table 1).

### 3.2 Overall connectome organization

There was no significant difference (*p* > .05) in overall connectivity strength, small‐wordness, and global efficiency between pre‐ and post‐session both in the treatment and the no‐treatment groups. For nodal metrics, no nodes survived the false discovery rate correction of *q* < .05. When the statistical level was set to *p* < .01, we found that the right insula showed an increase in nodal efficiency and nodal strength after nonpharmacological treatment (Figure S1).

### 3.3 Altered rich club organization

**FIGURE 2:**
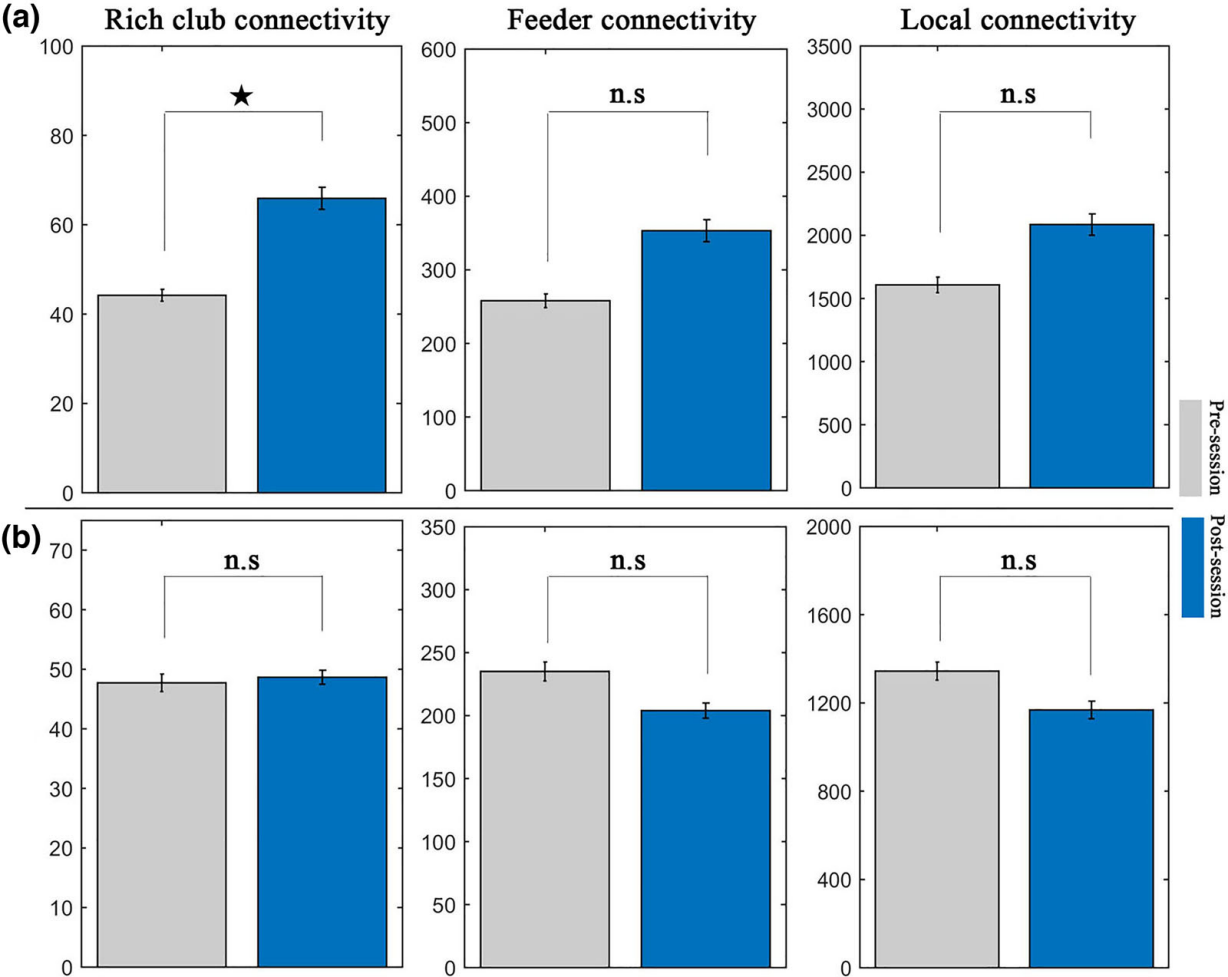
Statistical differences of rich club connectivity, feeder connectivity, and local connectivity between pre‐ and post‐session in treatment group (A) and no‐treatment group (B). The star meant that the difference was significant (*p* < .05). n.s, no significant.

**FIGURE 3:**
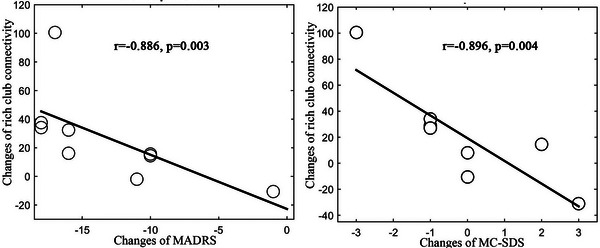
Significant negative correlation of changes of rich club connectivity (post‐session minus presession) with changes of Montgomery–Asberg Depression Rating Scale (MADRS) and Marlowe–Crowne Social Desirability Scale (MC‐SDS) (post‐session minus presession) in patients of treatment group.

In the treatment group, the rich club connectivity was significantly increased (*p* = .0107) after nonpharmacological treatment, whereas the feeder connectivity and local connectivity remained unchanged (*p* > .05) (Figure 2A). In the no‐treatment group, all of the rich club connectivity, feeder connectivity, and local connectivity remained unchanged (*p* > .05) (Figure 2B). There was a significantly negative correlation between rich club connectivity changes (post‐session minus presession) and MADRS score changes (post‐session minus presession: *r* = −.886, *p* = .003) and MC‐SDS score changes (post‐session minus presession: *r* = −.896, *p* = .004) (Figure 3).

### 3.4 Altered subnetwork

**FIGURE 4:**
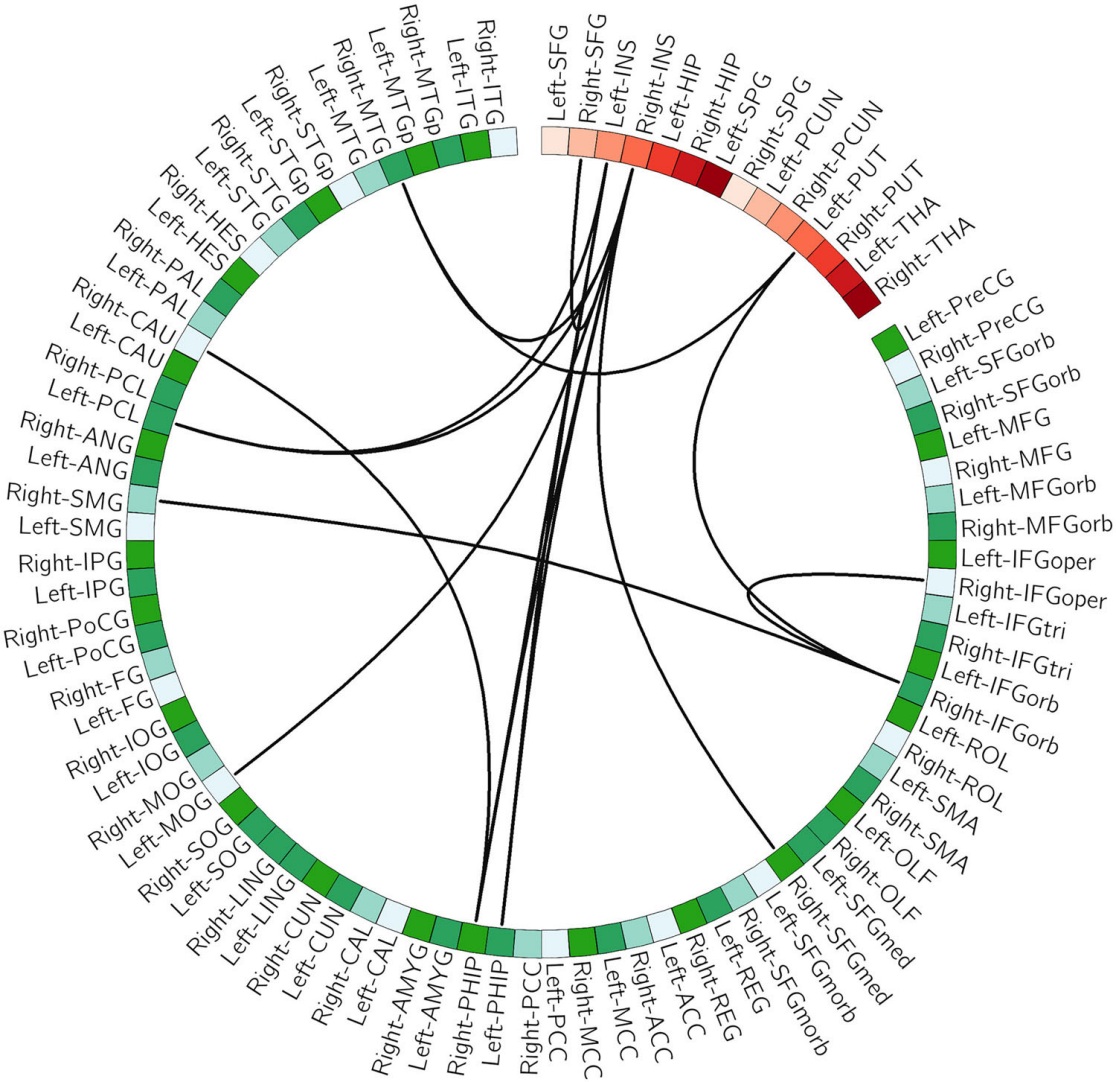
Enhanced functional connectivity (black line) following nonpharmacological treatment in patients with major depressive disorder (MDD). The red nodes donate rich club nodes, whereas the left nodes were peripheral nodes. The full name of these brain areas can be seen in Table S1.

NBS identified one subnetwork (*p* = .039), within which the FCs were significantly higher after nonpharmacological treatment. This subnetwork mainly consisted of connections between the insula cortex and parahippocampus, frontal and temporal cortex, and between putamen and frontal/temporal cortex (Figure 4, Table 2). By applying the subnetwork affiliations of the AAL atlas (Table S1), we found that the altered subnetwork included connections between SN and DMN, between SN and frontoparietal network (FPN), between SN and sensorimotor network (SMN), and between subcortical network (SCN) and DMN. NBS did not identify any significantly altered subnetworks in the no‐treatment group.

**TABLE 2:** Increased functional connectivity following nonpharmacological treatment revealed by network‐based statistic.

ROI 1	ROI 2	Mean value of presession	Mean value of post‐session	*t*‐Value (post‐session vs. presession)
Right INS	Right SFG	.15	.47	5.42
Right IFGorb	Right IFGoper	.54	.92	4.59
Right SMG	Right IFGorb	.28	.59	4.62
Left PUT	Right IFGorb	.06	.39	4.41
Right INS	Right SFGmed	.03	.35	4.52
Left PHIP	Left INS	.11	.34	3.98
Right PHIP	Left INS	.1	.36	4.05
Left PCL	Left INS	.36	.64	3.96
Left PHIP	Right INS	.04	.35	4.09
Right PHIP	Right INS	.08	.4	5.19
Left MOG	Right INS	.21	.41	4.08
Left PCL	Right INS	.29	.56	3.98
Right MTG	Right INS	.22	.54	4.31
Right CAU	Right PHIP	.07	.37	3.88
Right MTG	Left PUT	.2	.41	4.66

*Note*: The full name of these ROIs and subnetwork affiliations can be found in Table S1.

Abbreviations: INS, insula; PUT, putamen; ROI, regions of interest; SFG, superior frontal gyrus.

## 4 DISCUSSION

In the current study, we found that patients with MDD experienced remission of clinical symptoms and showed enhanced rich club connectivity after non‐pharmacological treatment. Moreover, the change in rich club connectivity was negatively correlated with the change of depressive symptoms. In addition, NBS revealed treatment‐related increase in FCs among SN, DMN, FPN, and SMN.

The phenomenon of “rich clubs” in brain networks originated from the discovery in the nematode worm Caenorhabditis elegans that some nodes connected more efficiently than others (Towlson et al., 2013). It was confirmed that these regions served as the intermediary of global information flow rather than isolated subnetworks, which contributed to the integration of neural information (Kim & Min, 2020). As the core architecture of the network, rich clubs played an important role in brain function control and communication of network modules (Liu et al., 2021). Now, many studies had found that rich club connections were abnormal in patients with mental disorders, such as schizophrenia (van den Heuvel et al., 2013), and patients with MDD (Liu et al., 2021).

Specifically, a previous study found that rich club connections in late‐life depression were significantly weakened compared to comparison controls (Mai et al., 2017). The connectivity strength of rich‐club connections in the precuneus was reduced in the subjective memory complaint individuals with depression compared with those without depression, which was correlated with the severity of depressive symptoms (Kim et al., 2019). After 8 weeks of pharmacological treatment, significantly enhanced rich club connections were observed in the patients (Yoon et al., 2016). However, other studies had not found significant differences in rich club connections between MDD and controls (He et al., 2022), and the discrimination before and after treatment was poor (Wang et al., 2019). The inconsistent results may be due to the type of depression, analysis methods, and medication. In the current study, we found that after nonpharmacological treatment, the rich club connectivity was increased. Moreover, there was a negative correlation between the rich club connectivity changes and clinical symptoms changes, indicating that the more increase in rich club connections, the more improvement of depressive symptoms. Therefore, we speculated that rich club may be related to global efficiency, and treatment can improve the brain efficiency of MDD patients, thus alleviating depressive symptoms. These results indicated that rich club can be used as a brain imaging marker for depressed patients and their therapeutic effects. On the other hand, some studies had also found reductions in feeder and local connections in depressed patients than controls (Liu et al., 2021; Mai et al., 2017). In addition, the feeder connectivity was associated with the remission of clinical symptoms in MDD patients after antidepressant treatment (Wang et al., 2019). However, we did not find significant changes in feeder connection in patients with MDD following treatment. This may be due to the different brain mechanisms between pharmacological and nonpharmacological treatments.

Our study further found that after nonpharmacological treatment, depressed patients showed increased connectivity of relevant brain circuits, mainly including the connectivity between SN and FPN, SN, and DMN, SN and SMN, and between SCN and DMN. Previous studies found that abnormal connectivity of brain functional networks mainly occurred in the DMN, SN, and FPN. These central hubs formed an extensive triple network that controlled individual cognitive and emotional functioning. The DMN, which was responsible for self‐reference and internally directed attention (Andrews‐Hanna et al., 2014), had been found to have hyperconnectivity in MDD patients (Kaiser et al., 2015) and reported as an indicator of increased rumination (Sheline et al., 2010). The FPN and SN were involved in attention cognitive control and emotion regulation (Snyder, 2013), and emotional processing and monitoring salient events (Choi et al., 2012), respectively. Previous studies found low connectivity of SN in MDD patients (Javaheripour et al., 2021), in which the anterior insular, as the main hub of SN, had a wide functional network that regulated the activities of DMN and FPN (Sridharan et al., 2008). Some researchers also found abnormal FC between SN and DMN, SN and FPN in this disease (Kaiser et al., 2015; Luo et al., 2021; Menon & Uddin, 2010; Zanto & Gazzaley, 2013). In addition, changes in FC of other networks, including SCN and SMN, had also been confirmed to be related to the pathophysiology of MDD (Brown et al., 2017; Rolls et al., 2018; Zeng et al., 2012). These studies indicated that MDD was a disorder in which brain networks were dysregulated.

Previous CBT studies found that the FCs within SN and between SN and DMN were decreased in patients with mild‐to‐moderate depression after treatment and were associated with improvement in clinical symptoms (Jacobs et al., 2016; Yang et al., 2018). In addition, the FC of the superior frontal gyrus in FPN was significantly increased after CBT treatment (Villa et al., 2020), which meant that treatment could normalize the disrupted FC (Ritchey et al., 2011; Shou et al., 2017). Studies of neurofeedback treatment could also normalize specific brain areas and brain network abnormalities, including SMN (Bezmaternykh et al., 2021), DMN (Buckner et al., 2008), and insula (Seeley et al., 2007). The increased FCs among SN, DMN, FPN, SMN, and SCN following treatment found in the current study were in accordant with previous findings, suggesting that the improvement of depressive symptoms might be associated with the interaction of resting‐state networks.

Several limitations should be addressed. First, the limited sample size might reduce the statistical power. Future studies are warranted to include more participants. In spite of this, we still found significantly increased FC following treatment. Second, the current study did not include a healthy control group, meaning that we cannot exclude the potential effect of normal brain development. Third, the treatment group mixed CBT and real‐time fMRI neurofeedback treatments. This meant that there was a potential hypothesis that the two treatment strategies shared common brain mechanisms, which was unclear at present. Future studies will include large datasets to test the hypothesis.

## 5 CONCLUSION

In conclusion, we found that after nonpharmacological treatment, the rich club connectivity of functional brain networks in patients with MDD was increased. The enhancement of rich club connectivity was negatively correlated with the improvement of depressive symptoms. Furthermore, the treatment‐related increase in FC was observed mainly among SN, DMN, FPN, SMN, and SCN. These findings suggested that the nonpharmacological treatment might target the rich club connectivity and increase the brain network efficiency, as a consequence of the remission of clinical symptoms.

## AUTHOR CONTRIBUTIONS

Zhiliang Long designed the study. Danni Chen and Zhiliang Long performed the data analysis and drafted the manuscript. Danni Chen, Zhiliang Long, and Xu Lei discussed and revised the manuscript.

## CONFLICT OF INTEREST STATEMENT

The authors declare that they have no conflicts of interest.

### PEER REVIEW

The peer review history for this article is available at https://publons.com/publon/10.1002/brb3.3198.

## Supporting information


**Figure S1** Increased nodal efficiency (left) and nodal strength (right) of right INS (red ball) after nonpharmacological treatment in MDD was found when the statistical level was set to be *p* < .01. The size of ball indicates significance. The larger the size, the more significance. MDD, major depressive disorder; INS, insular.

**Table S1** The abbreviation and full names of AAL atlas and their subnetwork affiliations.

## Data Availability

The database used in the current study was obtained from the online OpenNeuro dataset (https://openneuro.org/datasets/ds003007/versions/1.0.1).
