# Supplementary figures and images for: Enhanced rich club connectivity in mild or moderate depression after nonpharmacological treatment: A preliminary study
Source: Brain Behav. 2023 Sep 7;13(10):e3198. doi: 10.1002/brb3.3198 (PMC10570500; doi:10.1002/brb3.3198)

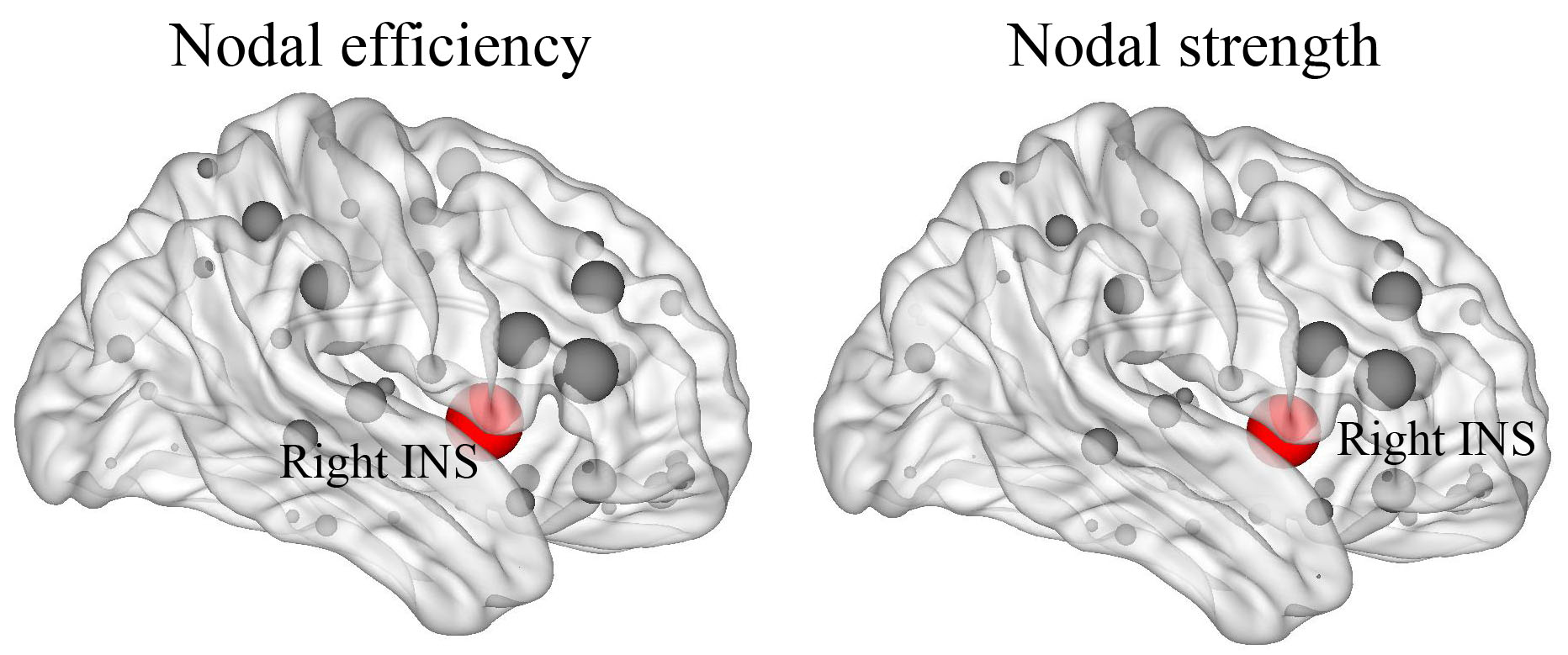

Supplement: Supplementary file 1 — Figure S1 Increased nodal efficiency (left) and nodal strength (right) of right INS (red ball) after nonpharmacological treatment in MDD was found when the statistical level was set to be p < .01. The size of ball indicates significance. The larger the size, the more significance. MDD, major depressive disorder; INS, insular. [file BRB3-13-e3198-s002.jpg]
