# Supplementary material for: Enhanced rich club connectivity in mild or moderate depression after nonpharmacological treatment: A preliminary study
Source: Brain Behav. 2023 Sep 7;13(10):e3198. doi: 10.1002/brb3.3198 (PMC10570500; doi:10.1002/brb3.3198)
Supplement: Supplementary file 2 — Table S1 The abbreviation and full names of AAL atlas and their subnetwork affiliations. [file BRB3-13-e3198-s001.docx]

**Table S1**. The abbreviation and full names of AAL atlas and their subnetwork affiliations

| Abbreviation | Full name of AAL regions | Subnetwork affiliation |
| --- | --- | --- |
| PreCG | Precentral gyrus | Sensorimotor |
| SFG | Superior frontal gyrus | Frontoparietal |
| SFGorb | Superior frontal gyrus,orbital part | Frontoparietal |
| MFG | Middle frontal gyrus | Salience/frontoparietal/attention |
| MFGorb | Middle frontal gyrus,orbital part | Frontoparietal |
| IFGoper | Inferior frontal gyrus,opercular part | Cingulo-opercular |
| IFGtri | Inferior frontal gyrus,triangular part | Salience/frontoparietal/attention |
| IFGorb | Inferior frontal gyrus,orbital part | None |
| ROL | Rolandic operculum | Auditory/cingulo-opercular |
| SMA | Supplementary motor area | Sensorimotor |
| OLF | Olfactory cortex | None |
| SFGmed | Medial Superior frontal gyrus | Default-mode |
| SFGmorb | Medial orbital frontal gyrus | Default-mode |
| REG | Gyrus rectus | None |
| INS | Insula | Salience/cingulo-opercular |
| ACC | Anterior cingulate gyri | Default-mode/salience |
| MCC | Median cingulate gyri | Salience/cingulo-opercular |
| PCC | Posterior cingulate gyrus | Default-mode |
| HIP | Hippocampus | None |
| PHIP | Parahippocampal gyrus | Default-mode |
| AMYG | Amygdala | None |
| CAL | Calcarine fissure | Visual |
| CUN | Cuneus | Visual |
| LING | Lingual gyrus | Visual |
| SOG | Superior occipital gyrus | Visual |
| MOG | Middle occipital gyrus | Visual |
| IOG | Inferior occipital gyrus | Visual |
| FG | Fusiform gyrus | Visual |
| PoCG | Postcentral gyrus | Sensorimotor |
| SPG | Superior parietal gyrus | Salience/attention |
| IPG | Inferior parietal gyri | Frontoparietal/attention |
| SMG | Supramarginal gyrus | Auditory/cingulo-opercular |
| ANG | Angular gyrus | Default-mode |
| PCUN | Precuneus | Default-mode |
| PCL | Paracentral lobule | Sensorimotor |
| CAU | Caudate nucleus | Subcortical |
| PUT | Putamen | Subcortical |
| PAL | Pallidum | Subcortical |
| THA | Thalamus | Subcortical |
| HES | Heschl gyrus | Auditory |
| STG | Superior temporal gyrus | Auditory/attention |
| STGp | Superior temporal gyrus, temporal pole | cingulo-opercular |
| MTG | Middle temporal gyrus | Default-mode |
| MTGp | Middle temporal Temporal pole | Default-mode |
| ITG | Inferior temporal gyrus | None |

Note that some ROIs were assigned into more than one subnetwork. ROI, regions of interest; AAL: Automated Anatomical Labeling.
